# Mediating Effect of Mutuality on Health-Related Quality of Life in Patients with Parkinson's Disease

**DOI:** 10.1155/2018/9548681

**Published:** 2018-09-16

**Authors:** Michaela Karlstedt, Seyed-Mohammad Fereshtehnejad, Dag Aarsland, Johan Lökk

**Affiliations:** ^1^Karolinska Institutet, Department of Neurobiology Care Sciences and Society, Division of Clinical Geriatrics, Floor 7 141 83 Huddinge, Stockholm, Sweden; ^2^Department of Neurology and Neurosurgery, McGill University, Montreal, QC, Canada; ^3^Karolinska Institutet, Alzheimer Disease Research Center (KI-ADRC) Novum, Floor 5 SE-141 86, Stockholm, Sweden; ^4^Department Old Age Psychiatry, Kings College, London, UK

## Abstract

The relationship quality, mutuality, has been identified as a protective factor in family care situations, but its role in mediating health-related quality of life (HRQoL) in patients having Parkinson's disease (PD) is not known. Data on patients' and partners' mutuality (MS), motor signs (UPDRS III), non-motor symptoms (NMSQuest), impaired cognition (IQCODE), dependency in activities of daily life (ADL), and HRQoL (PDQ8) were collected from 51 dyads. Structural equation model with manifest variables was applied to explore if the MS score mediated the effect of UPDRS III, NMSQuest, IQCODE, and dependency in ADL on PDQ8. The results suggest that increasing severity of motor and non-motor symptoms decreases patients' mutuality which leads to worse HRQoL. Partners' mutuality mediated the effect of impaired cognition which in turn decreased patients' mutuality. The findings enhance our understanding of how various symptoms may influence PD patients' HRQoL. This may help clinicians to personalize interventions to provide more effective interventions to improve the lives of patients with PD.

## 1. Introduction

Parkinson's disease (PD) is a complex disorder which often influences several aspects of daily life. It is well known that the combination of motor impairment and a wide variety of non-motor symptoms (NMS) interferes with daily activities and can contribute to impaired health-related quality of life (HRQoL) [1–6]. Living with a chronic condition can invoke many changes in a couple and disrupt social interactions and connectedness [7, 8]. PD patients commonly rely on their partners who often assist them with managing their health. This can lead to an imbalance of the support one receives or gives, resulting in a change of roles and relational dynamics within the dyads [9, 10]. The positive quality of the relationship, defined as mutuality, has been described as having four dimensions: love and affection, shared pleasurable activities, shared values, and reciprocity [11, 12]. In other words, mutuality refers to the quality of the interaction between persons, here a PD patient and a spouse, and involves feelings of closeness, reciprocity of sentiment, understanding of one another, and shared goals and activities. Growing evidence from caregiving research suggests that high mutuality of caregivers is associated with high emotional well-being and acts as a protective factor of negative caregiving outcomes. A review has also shown that mutuality may decrease over the course of a chronic condition [13]. However, research on perceived mutuality of PD patients is scarce and mainly based on small sample sizes. Ricciardi et al. found PD patients to be more depressed and less satisfied with their marriage than their partners [14]. Insecurity and concern if the partner will stay in the relationship or start to resent them as PD advances are feelings that also have been expressed by PD patients in a qualitative interview study [9]. Despite these negative effects, Mavandadi et al. in a small cross-sectional study found an association between greater marriage quality and perceived benefits or personal growth from having PD [15]. Understanding the interaction between stressors, mediators, and health outcomes often accompanying PD may pave the way for care models and interventions that improve well-being and HRQoL. Guided by the proposed conceptual stress model for individuals with dementia, the aim of the present study was to explore if mutuality acts as a mediator on PD patients' HRQoL [16]. Mediation analysis is often used to test theories regarding a process [17]. In statistics, a mediation model is designed to explain the mechanism that underlies an observed relationship between an independent variable (here PD related symptoms) and a dependent variable (here HRQoL) via the inclusion of a third hypothetical variable, known as a mediator (here mutuality). Rather than a direct causal association, mediation proposes that the independent variable affects the mediator variable, which in turn influences the dependent variable [18].

According to most of the stress process theories, health outcomes are influenced by primary stressors which often refer to disease-related factors or the individuals' appraisal of the situation. These primary stressors can have a direct or indirect effect on health outcomes through different strains (e.g., self-esteem and role strain) or protective factors such as mutuality [16, 19, 20]. In mediation analysis, primary stressors are seen as antecedents of mediators and health outcome variables [17]. We recently showed that primary stressors such as motor and NMS were adversely associated with PD patients' mutuality and PD patients' HRQoL [21] and that patients' mutuality was positively associated with HRQoL, indicating that mutuality may act as a mediator. Also, partners' mutuality was positively associated with patients' mutuality, indicating that partners' mutuality may act as a mediator between significant stressors and patients' mutuality or patients' HRQoL [21]. To our knowledge, there is no published study exploring if mutuality acts as a mediator on PD patients' HRQoL. By testing the mediating effect of mutuality, we will expand our previous research and disentangle different pathways that could explain the effect of PD specific symptoms on patients' HRQoL. Furthermore, the results may also provide new knowledge if mutuality is an effective mechanism to improve PD patients' HRQoL. Guided by the aforementioned theoretical frameworks, we hypothesized that motor symptoms, NMS, impaired cognition, and dependency in ADL act as primary stressors with direct or indirect effects mediated through patients' mutuality and partners' mutuality on patients' HRQoL.

## 2. Materials and Methods

### 2.1. Participants

For this cross-sectional study, 51 patients with mild to moderate PD and their partners were recruited through movement disorders clinics at Karolinska University Hospital and through advertisement in the journal of the Swedish Parkinson's Disease Association. The dyads had a well-established relationship and had been living together, on average, for 38.4 years (SD = 14.59). Neither of the partners were employed as caregivers nor did the dyads rear small children. More details are published elsewhere [21]. The study was approved by the local research ethics committee in Stockholm, Sweden (registration number: 2013/1812-31/3), and was conducted in accordance with the Declaration of Helsinki.

### 2.2. Measurement

To evaluate severity of PD specific motor signs, the 14-item Unified Parkinson's Disease Rating Scale-Part III (UPDRS III) was used. The scale is answered using a 5-point Likert scale. Higher scores indicate more severe motor signs [22].

To detect PD specific non-motor manifestations in domains such as urinary, cardiovascular, depression/anxiety, memory, sexual function, sleep disorder, digestive, hallucination/delusion, and miscellany, the Non-motor Symptoms Questionnaire (NMSQuest) was used. The scale comprises 30 items scored “yes” or “no.” Higher scores indicate higher frequency of non-motor manifestations [23, 24].

The Informant Questionnaire on Cognitive Decline in the Elderly (IQCODE) was used to assess functional changes associated with cognitive status in the patients. The scale is answered using a 5-point Likert scale and comprises 26 items. The individual scores are ranging between 1 and 5 and are calculated by the mean across all item scores. Higher scores (>3) indicate a decline in cognitive functioning. The questionnaire was filled out by the partner. For this scale, Cronbach's alpha has been reported ranging from 0.93 to 0.97 in several studies [25].

The patient's level of dependency in activities of daily life was assessed using a modified form of the extended Katz index [26]. The scale contains items assessing grooming/dressing, bathing, food intake, toileting, walking/transferring, housekeeping, and shopping (0 = need no help to 3 = need all help). The scale was filled out by the partner. A dichotomous variable (0 = independent; 1 = dependent) was created aiming to assess dependency.

The 8-item Parkinson's Disease Questionnaire-short form (PDQ8) was used to measure PD specific HRQoL. The scale covers domains such as mobility, activities of daily life, emotional well-being, stigma, social support, cognitions, communication, and bodily discomfort. The scale is answered using a 5-point Likert scale. A summary index was calculated ranging from 0 to 100. Higher scores indicate worse HRQoL [27].

The 15-item mutuality scale (MS) was used to measure the positive quality of the caregiver-care receiver relationship [11, 12]. The scale is answered using a 5-point Likert scale (0 = not at all to 4 = a great deal). It covers domains such as love and affection (3 items), shared pleasurable activates (4 items), shared values (2 items), and reciprocity (6 items). The individual scores are ranging between 0 and 4 and are calculated by the mean across all item scores. Higher scores indicate higher quality of the mutual relationship between the care-dyads. For the Swedish version of MS, Cronbach's alpha was calculated as 0.936 for PD patients in MS and as 0.933 for PD partners in MS [28].

### 2.3. Statistical Analysis

Characteristics of the included PD dyads were described using frequency, percentage, means (*m*), and standard deviation (SD).

Two of the participants had one single missing item each within the NMSQuest scale. The individual scores were larger than the sample median. To avoid case-wise deletion and loss of power, these items were imputed with a zero score. To calculate ranking of each NMSQuest domain, the sum of positive responses in each domain was divided by the maximum possible positive responses in the corresponding domain.

**Figure 1:**
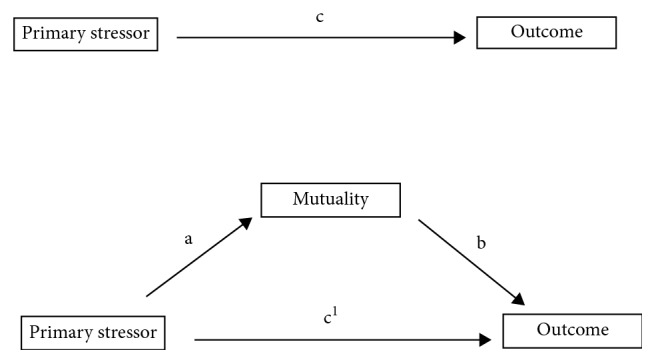
Illustration of a simple mediation with total, direct, and indirect effects.

To test our mediation hypotheses, structural equation modeling (SEM) with manifest variables was performed.  Figure 1 illustrates a schematic model of a simple mediation [17]. At the top in Figure 1, the total effect (path c) can be described as the sum of direct and indirect effects of the primary stressor on the outcome variable or simplified as the effect without the mediator in the equation. Path a (at the bottom in Figure 1) represents the primary stressor's effect on the mediator controlling for the effect of the mediator on the outcome variable (path b). The same applies for path b, which represents the mediator's effect on the outcome variable. The indirect effect is usually calculated as the product of a × b. The direct effect (path c^1^) can be described as the effect between the primary stressor and the outcome controlling for the indirect effect [17].

Prior to the analyses, assumptions of multicollinearity were examined through tolerance and variance inflation factor (VIF (1/tolerance)). Tolerance (>0.4) and VIF index (<2.5) were considered acceptable. No influential multivariate outliers were detected using the Mahalanobis and Cooks distance [29]. Based on our prior study and the hypothesis we generated, UPDRS III, NMSQuest, IQCODE, and ADL served as primary stressors (exogenous variables), while patients' HRQoL (PDQ8) served as the outcome variable (endogenous variable) and patients' mutuality and partners' mutuality served as mediators (endogenous variables).

The fit of the models was tested using the Chi-square test, Comparative fit index (CFI), Normed fit index (NFI), Tucker–Lewis index (TLI), Goodness-of-fit statistic (GFI), and the Root-mean-square error of approximation (RMSEA). A model was considered well fitted when the chi-square value was non-significant, TLI, CFI, NFI, and GFI > 0.95, and RMSEA < 0.05 [30]. The square multiple correlation was used to assess how much of the variance in mutuality and HRQoL was explained by the included exogenous variables.

Total, direct, and indirect effects between exogenous and endogenous variables were calculated using maximum likelihood estimation and are presented as standardized path coefficients. An advantage of SEM is that direct and indirect effects (mediation) can be tested simultaneously within the model. To test the indirect effects, the bias-corrected bootstrap method was used [31]. The 95% confidence interval (CI) was determined following 2000 iterations from the sample of 51 participants.

To classify and understand different types of mediation, the proposed typology and interpretation of mediation by Zhao et al. was used [32]. A complementary mediation is when both indirect and direct effects exist and point in the same direction, similar to what Baron and Kenny referred to as partial mediation [32, 33]. The second type of mediation, named competitive mediation, is when both the indirect and direct effects exist but the effects point in opposite direction, which also has been referred in the literature as inconsistent mediation [32, 34]. The third type of mediation, named as indirect-only mediation, is when indirect effect exists but there is no direct effect, referred to as full mediation by Baron and Kenny [32, 33]. A complementary mediation or a competitive mediation indicates that there may be omitted mediators which coexist with the mediator within the explored model. An indirect-only mediation implies that the mediator fully explains the association between the included variable and the outcome variable. Two types of patterns consistent with non-mediation are also described, namely, direct-only non-mediation when direct effects between the independent variable and the outcome exist but there is no indirect effect and no-effect non-mediation when neither direct nor indirect effects exist [32].

In the whole analysis, the path model was adjusted by age and gender. Based on prior results, gender was chosen to adjust the effect on PD patients' mutuality [21]. A *p* value of 0.05 or less was regarded as statistically significant.

All data analyses were conducted using SPSS Statistics for Windows, version 23 (IBM Corp., Armonk, NY, USA), and AMOS graphics module version 23 (IBM INC).

## 3. Results

### 3.1. Participants

The mean age of patients and partners was 70.9 (SD = 8.5) and 70.7 (SD = 9.3) years, respectively. Of the patients, 35/51 (68.6%) needed some form of supervision or help from their partners in daily activities. Other demographic and clinical characteristics are presented in Table 1.

All patients were treated with a combination of antiparkinsonian drugs. Of the 51 patients, four were treated with deep brain stimulation, three with carbidopa-levodopa infusion, and two with infusion of dopamine agonists. Complications were quite common: 33/48 (65%) had experienced dyskinesia and 29/48 (57%) had motor fluctuations. Urinary problems (76%) were the most frequent reported non-motor domain, and hallucination/delusion (21%) was the least reported domain (Table 2).

### 3.2. Path Analysis

**Figure 2:**
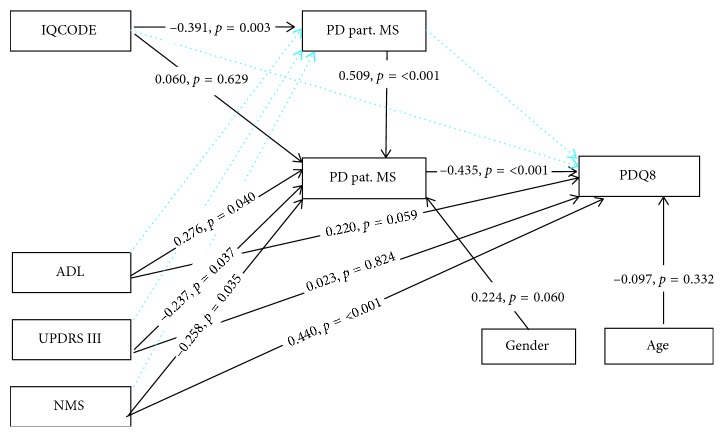
Direct effects reported as standardized path coefficients for the final model with HRQoL (PDQ8) as the outcome variable. Dashed lines are nonsignificant direct paths which were removed in the final model. The best fit of the final path model was achieved with *χ*^2^* *=* *7.980, *df* =* *9, CMIN/DF* *=* *0.887, *p*=0.536, GFI* *=* *0.968, NFI* *=* *0.939, CFI* *=* *1.0, TLI* *= 1.0, and RMSEA* *=* *0.00 (95% CI* *=* *0.00 − 0.146) (*n*=51 dyads). Note: PD: Parkinson's disease; Pat. MS: PD patients' mutuality scale; Part. MS: PD partners' mutuality scale; PDQ8: Parkinson's disease questionnaire summary index; IQCODE: informant questionnaire on cognitive decline in the elderly; NMS: non-motor symptoms questionnaire; UPDRS III: unified Parkinson's disease rating scale-part III; ADL: activities of daily life (0 = independent; 1 = dependent); gender: 0 = female; 1 = male; age: PD partners' age.

Figure 2 illustrates the relationship of the included factors which affects patients' and partners' mutuality and patients' HRQoL.

The first model resulted in acceptable fit. However, several of the path coefficients were small and non-significant including the path between partners' MS score and PDQ8 (beta = −0.027; *p*=0.825), indicating that partners' mutuality did not act as a mediator on patients' HRQoL. Due to the small sample size, all unrequired and non-significant paths were discarded one by one (Figure 2). The final model resulted in acceptable fit. The fit of the final model and the standardized direct path and coefficients are presented in Figure 2. The final model explained 15.3% of the variance in partners' mutuality, 42.0% in patients' mutuality, and 55.8% in patients' HRQoL.

### 3.3. Direct Effects

The significant direct effect of patients' MS score (beta = −0.435; *p* < 0.001) on PDQ8 indicated that patients' mutuality may act as a mediator between the included clinical variables, which were significantly associated with patients' mutuality (Figure 2): UPDRS III (beta = −0.237; *p*=0.037), NMS (beta = −0.258; *p*=0.035), and ADL (beta = 0.276; *p*=0.040). This means that increasing severity of motor and NMS was associated with a lower level of patients' mutuality. Furthermore, a higher level of patients' mutuality was associated with better HRQoL, and the combined effect of these symptoms and mutuality may influence patients' HRQoL. Patients who had some form of dependency in ADL, assessed by the partners, had higher MS scores compared to the non-dependent patients. Impaired cognition was not associated with the patients' MS scores (beta = 0.060; *p*=0.629). Instead, worse cognition (beta = −0.391; *p*=0.003) decreased partners' MS scores. Furthermore, increasing MS scores of partners (beta = 0.509; *p* < 0.001) had a positive direct effect on patients' MS scores. This means that the effect of reduced cognitive function may influence patients' mutuality through partners' mutuality.

### 3.4. Indirect Effect and Total Effect

Indirect effects and total effects are presented in Table 3.

The mediating test of indirect effects revealed that the effect of NMS (beta = 0.112; *p*=0.043) on patients' HRQoL was mediated by patients' mutuality, implying that increasing frequency of NMS leads to a decrease in patients' mutuality, in turn leading to worse HRQoL (increasing PDQ8 score). The significant direct (beta = 0.440; *p*=0.001) and total effects (beta = 0.552; *p*=0.001) of NMS on HRQoL indicate a complementary mediation and point to the possibility of omitted mediators.

The effect of increasing UPDRS III scores (beta = 0.103; *p*=0.026) on patients' HRQoL was also mediated by patients' mutuality. In other words, increasing severity of motor symptoms decreases patients' mutuality resulting in worse HRQoL. There was no significant direct (beta = 0.023; *p*=0.883) or total effect (beta = 0.126; *p*=0.372) of increasing UPDRS III scores on patients' HRQoL signaling an indirect-only mediation.

Patients' mutuality did not mediate the effect of impaired cognition. Instead, partners' mutuality mediated the effect of increasing IQCODE scores (beta = −0.199; *p*=0.011) on patients' mutuality. In other words, worse cognition decreases partners' mutuality, in turn leading to the decreasing level of patients' mutuality. The lack of significant direct effect (beta = 0.060; *p*=0.629) points to an indirect-only mediation.

## 4. Discussion

This is, to our knowledge, the first study to explore if mutuality of PD patients and PD partners acts as a mediator between clinical PD features and patients' HRQoL. Our findings suggest that patients' mutuality mediates the effect of motor and NMS on patients' HRQoL. In contrast to our initial hypothesis, partners' mutuality did not act as a mediator on patients' HRQoL. Instead, partners' mutuality mediated the effect of impaired cognition on patients' mutuality.

We explored direct and indirect effects of specific PD symptoms on patients' HRQoL. Consistent with prior research studies, NMS had a larger direct negative impact on patients' HRQoL than motor symptoms [2, 3, 21, 35–37]. Our findings suggest that the effect of NMS on HRQoL was also mediated by patients' mutuality, and this type of indirect effect could be classified as a complementary mediation [32]. Although there might be other important mediators such as personality, coping, and perceived external support, the combined effect of NMS and mutuality on HRQoL has an important contribution [16]. The mean frequency of NMS was 12 which is similar to that in other studies [23, 38, 39]. Urinary problems (76%), cardiovascular (44%), depression/anxiety (43%), memory (42%), and sexual dysfunction (42%) were the most frequent reported non-motor domains. Thus, consequences of the wide variety of NMS are likely to influence several domains of mutuality such as love and affection, less-shared leisure activities with the partner, and perhaps disagreement in how to adjust and cope with PD. This can cause tension and result in a less supportive relationship leading to worse HRQoL. This corresponds with results from a qualitative study where PD patients expressed that family members do not understand how anxiety, depression, and apathy influence daily activities [40].

Indirect-only mediation was identified for the effect of motor symptoms on patients' HRQoL and patients' mutuality. The indirect-only mediation indicates that the motor symptoms' influence on HRQoL is only effective through motor symptoms' effect on patients' mutuality. This means that increasing severity of motor symptoms did not directly influence patients' HRQoL, instead, the combination of motor symptoms and mutuality was associated with worse HRQoL. This finding corresponds with results from a qualitative study where motor symptoms and constant struggle with unpredictability made the patients engage in fewer leisure activities and in some cases feel alone and less close to their partner [9]. Similarly, a recent study found that severity of motor features such as UPDRS III, falls, and ADL were mediated by NMS such as depression, psychosocial functioning, and nutritional status which led to worse HRQoL [36].

Impaired cognition has a detrimental effect on patients' HRQoL [3, 6, 41]. However, in the present study, impaired cognition was not significantly associated with patients' mutuality or HRQoL. Instead, an indirect-only mediation of impaired cognition on patients' mutuality was observed through partners' mutuality, indicating that worsening of cognitive function decreases partners' mutuality, which in turn leads to a lower level of patients' mutuality. The non-significant direct and indirect effects of impaired cognition on patients' HRQoL may be explained by the fact that cognitive function was assessed by partners and not the patients themselves. Another explanation may be that the cognitive decline was mild and the decline did not influence patients' appraisal of daily functioning. Thus, patients' perceived cognitive function and its consequences were not in concordance with the assessment done by the partners.

Our findings suggest that patients' mutuality is a mediator between symptoms and HRQoL in PD and that partners' mutuality mediates the relationship between impaired cognition and patients' mutuality.

These findings can be helpful for clinicians. Understanding the complexity and the combined effect that PD symptoms and mutuality have on HRQoL may aid clinicians to identify highrisk dyads. Clinicians should discuss with PD dyads how PD affects different dimensions of mutuality. Setting regular family meetings, improving the knowledge of partners towards the motor and NMS of PD and their progression over time, as well as highlighting the importance of the dyadic relationship should be considered to enhance mutuality and consequently improve patients' quality of life. For example, if the patient no longer is able to engage in earlier joint pleasurable activities with their partner, as a result of either motor or NMS, interventions aiming to find new enjoyable activities may improve the patient's mutuality and HRQoL. Furthermore, changes in cognitive function may negatively affect reciprocity and relational roles. Interventions aiming to understand the others' perspective of how different symptoms influence different dimensions of mutuality may enhance understanding of one another and facilitate coping and adjustment to PD. Not all relational issues can be solved by clinicians, and couple therapy or counseling may be needed for those with low mutuality before the PD diagnosis or for those who are uncertain if they should remain in the relationship. Nevertheless, our results could help clinicians to personalize interventions and improve PD dyads' ability to cope with the challenges they may encounter. Although specific PD symptoms are not often explicitly defined in qualitative studies in general, it seems that PD either brings dyads together or creates a distance between the members of the dyad. Some dyads even seem to have succeeded to move from distance towards a closer relationship by working together and find solution to PD challenges [9, 10, 42].

Our findings should be interpreted with caution. The design was based on a stress process model for persons with dementia rather than PD [16], and the model is based on complex interrelationships between different factors that have not been explored in the present study. Future research should explore other potential mediators such as external support, perceived stress, or perceived dependency. Another limitation is that dependency was assessed by the partners rather than as perceived by the patients, which may contribute to the nonsignificant direct and indirect effects. Other limitations are the cross-sectional design and the relatively small sample size for SEM. Thus, conclusions regarding causality cannot be made. Also, the sample had a predominance of older patients with mild to moderate PD which limits the generalizability. Future research would benefit from using a larger sample consisting of PD patients with different severity stages and using a longitudinal design. Nonetheless, our findings provide novel insights into the association between clinical symptoms and HRQoL in PD and offer a basis for future research to further understand the complexity and experience of living with PD, thus helping health professionals improve the quality of lives of PD patients and their carers.

## Figures and Tables

**Table 1:** Sociodemographic and clinical features (*n*=51 dyads).

	Patient	Partner
Female, *n* (%)	22 (43.1)	29 (56.9)

Retired, *n* (%)	45 (88.2)	39 (76.5)

Working,^*∗*^*n* (%)	10 (19.6)	16 (31.4)

*Level of education,n(%)*		
Elementary	8 (15.7)	6 (11.8)
Secondary	11 (21.6)	16 (31.4)
University	32 (62.7)	29 (56.9)

*Level of income (SEK)*		
0–199000	13 (25.5)	13 (25.5)
200000–450000	27 (52.9)	30 (58.8)
>450000	11 (21.6)	8 (15.7)

MS, *m* (SD)	3.2 (0.65)	2.9 (0.77)
PD duration, *m* (SD)	8.4 (6.4)	—
UPDRS III, *m* (SD)	18.1 (5.8)	—
NMSQuest, *m* (SD)	12.1 (4.6)	—
IQCODE, *m* (SD)	3.2 (0.53)	—
PDQ8, *m* (SD)	27.4 (14.6)	—

*Dependency in ADL (n*=35)		
Shopping, *n* (%)	32 (91.4)	—
Cooking/cleaning, *n* (%)	28 (80.0)	—
Walking/transferring, *n* (%)	23 (65.7)	—
Bath/showering, *n* (%)	13 (37.1)	—
Grooming/dressing, *n* (%)	11 (31.4)	—
Toileting, *n* (%)	9 (25.7)	—
Food intake, *n* (%)	7 (20.0)	—

Note: PD: Parkinson's disease; MS: mutuality scale; PDQ8: Parkinson's disease questionnaire summary index; IQCODE: informant questionnaire on cognitive decline in the elderly; NMSQuest: non-motor symptoms questionnaire; UPDRS III: unified Parkinson's disease rating scale-part III; ADL: activities of daily life; ^*∗*^some of the study subjects were still working.

**Table 2:** Frequency of positive answers classified by NMSQuest^*∗*^ domains (*n*=51).

	Positive answers			
NMSQuest^*∗*^ domains	Number of items	Frequency	Maximum of possible	% of maximum
Urinary	2	78	102	76
Cardiovascular	2	45	102	44
Depression/anxiety	2	44	102	43
Memory	3	65	153	42
Sexual function	2	43	102	42
Sleep disorder	5	100	255	39
Digestive	7	135	357	38
Miscellany	5	88	255	35
Hallucination/delusion	2	21	102	21

^*∗*^NMSQuest = non-motor symptoms questionnaire.

**Table 3:** Indirect and total effects of disease-related factors on PD patients' health-related quality of life (bootstrap sample = 2000).

Effects	Path	Standardized path coefficient	95% CI bias-corrected percentile			*p* value
Indirect effect with one mediator	ADL ⟶ Pat. MS ⟶ PDQ8	−0.120	−0.300	—	0.014	0.079
	UPDRS III ⟶ Pat. MS ⟶ PDQ8	0.103	0.010	—	0.239	0.026
	NMS ⟶ Pat. MS ⟶ PDQ8	0.112	0.006	—	0.263	0.043
	IQCODE ⟶ Part. MS ⟶ Pat. MS	−0.199	−0.339	—	−0.057	0.011

Total effect	ADL ⟶ PDQ8	0.100	−0.115	—	0.351	0.335
	UPDRS III ⟶ PDQ8	0.126	−0.116	—	0.334	0.372
	NMS ⟶ PDQ8	0.552	0.344	—	0.735	0.001
	IQCODE ⟶ PD-pat. MS	−0.139	−0.343	—	0.157	0.295

Note: PD: Parkinson's disease; ADL: activities of daily life (0 = independent; 1 = dependent); Pat. MS: PD patients' mutuality scale; PDQ8: Parkinson's disease questionnaire summary index; UPDRS III: unified Parkinson's disease rating scale-part III; NMS: non-motor symptoms questionnaire; IQCODE: informant questionnaire on cognitive decline in the elderly; Part. MS: PD partners' mutuality scale.

## Data Availability

Due to ethical restrictions, raw data are not suitable for public deposition. Data are available upon request for the researcher who meets the criteria for access to confidential data.

## References

[1] Martinez-Martin P. (2017). What is quality of life and how do we measure it? Relevance to Parkinson’s disease and movement disorders. *Movement Disorders*.

[2] Muller B., Assmus J., Herlofson K., Larsen J. P., Tysnes O. B. (2013). Importance of motor vs. non-motor symptoms for health-related quality of life in early Parkinson’s disease. *Parkinsonism and Related Disorders*.

[3] Duncan G. W., Khoo T. K., Yarnall A. J. (2014). Health-related quality of life in early Parkinson’s disease: the impact of nonmotor symptoms. *Movement Disorders*.

[4] Fereshtehnejad S. M. (2016). Strategies to maintain quality of life among people with Parkinson’s disease: what works?. *Neurodegenerative Disease Management*.

[5] Aarsland D., Kramberger M. G. (2015). Neuropsychiatric symptoms in Parkinson’s disease. *Journal of Parkinson’s Disease*.

[6] Schrag A., Jahanshahi M., Quinn N. (2000). What contributes to quality of life in patients with Parkinson’s disease?. *Journal of Neurology, Neurosurgery, and Psychiatry*.

[7] Lyons R. F., Sullivan M. J. L., Ritvo P. G. (1995). *Relationships in Chronic Illness and Disability*.

[8] Soleimani M. A., Negarandeh R., Bastani F., Greysen R. (2014). Disrupted social connectedness in people with Parkinson’s disease. *British Journal of Community Nursing*.

[9] Martin S. C. (2016). Relational issues within couples coping with Parkinson’s disease: implications and ideas for family-focused care. *Journal of Family Nursing*.

[10] Smit L. J., Shaw R. L. (2017). Learning to live with Parkinson’s disease in the family unit: an interpretative phenomenological analysis of well-being. *Medicine, Health Care, and Philosophy*.

[11] Archbold P. G., Stewart B. J., Greenlick M. R., Harvath T. (1990). Mutuality and preparedness as predictors of caregiver role strain. *Research in Nursing & Health*.

[12] Archbold P., Greenlick M. R., Harvath T. A., Funk S. G., Tornquist E. M., Champagne M. T. (1992). The clinical assessment of mutuality and preparedness in family caregivers to frail older people. *Key Aspects of Elder Care: Managing Falls, Incontinence, and Cognitive Impairment*.

[13] Park E. O., Schumacher K. L. (2014). The state of the science of family caregiver-care receiver mutuality: a systematic review. *Nursing Inquiry*.

[14] Ricciardi L., Pomponi M., Demartini B. (2015). Emotional awareness, relationship quality, and satisfaction in patients with Parkinson’s disease and their spousal caregivers. *Journal of Nervous and Mental Disease*.

[15] Mavandadi S., Dobkin R., Mamikonyan E., Sayers S., Have T. T., Weintraub D. (2014). Benefit finding and relationship quality in Parkinson’s disease: a pilot dyadic analysis of husbands and wives. *Journal of Family Psychology*.

[16] Judge K. S., Menne H. L., Whitlatch C. J. (2010). Stress process model for individuals with dementia. *Gerontologist*.

[17] Rucker D. D., Preacher K. J., Tormala Z. L., Petty R. E. (2011). Mediation analysis in social psychology: current practices and new recommendations: mediation analysis in social psychology. *Social and Personality Psychology Compass*.

[18] MacKinnon D. P. (2008). *Introduction to Statistical Mediation Analysis*.

[19] Goldsworthy B., Knowles S. (2008). Caregiving for Parkinson’s disease patients: an exploration of a stress-appraisal model for quality of life and burden. *Journals of Gerontology Series B, Psychological Sciences and Social Sciences*.

[20] Greenwell K., Gray W. K., van Wersch A., van Schai P., Walker R. (2015). Predictors of the psychosocial impact of being a carer of people living with Parkinson’s disease: a systematic review. *Parkinsonism and Related Disorders*.

[21] Karlstedt M., Fereshtehnejad S. M., Aarsland D., Lokk J. (2017). Determinants of dyadic relationship and its psychosocial impact in patients with Parkinson’s disease and their spouses. *Parkinson’s Disease*.

[22] Fahn S., Elton R., Fahn S., Marsden C. D., Goldstein M. (1987). The unified Parkinson’s disease rating scale. *Recent Developments in Parkinson’s Disease*.

[23] Chaudhuri K. R., Martinez-Martin P., Schapira A. H. (2006). International multicenter pilot study of the first comprehensive self-completed nonmotor symptoms questionnaire for Parkinson’s disease: the NMSQuest study. *Movement Disorders*.

[24] Martinez-Martin P., Schapira A. H., Stocchi F. (2007). Prevalence of nonmotor symptoms in Parkinson’s disease in an international setting; study using nonmotor symptoms questionnaire in 545 patients. *Movement Disorders*.

[25] Jorm A. F. (2004). The informant questionnaire on cognitive decline in the elderly (IQCODE): a review. *International Psychogeriatrics*.

[26] Asberg K. H., Sonn U. (1989). The cumulative structure of personal and instrumental ADL. A study of elderly people in a health service district. *Scandinavian Journal of Rehabilitation Medicine*.

[27] Jenkinson C., Fitzpatrick R., Peto V., Greenhall R., Hyman N. (1997). The PDQ-8: development and validation of a short-form Parkinson’s disease questionnaire. *Psychology and Health*.

[28] Karlstedt M., Fereshtehnejad S. M., Winnberg E., Aarsland D., Lokk J. (2017). Psychometric properties of the mutuality scale in Swedish dyads with Parkinson’s disease. *Acta Neurologica Scandinavica*.

[29] Tabachnick B. G. (2012). *Using Multivariate Statistics*.

[30] Hooper D., Coughlan J., Mullen M. (2008). Structural equation modelling: guidelines for determining model fit. *Electronic Journal of Business Research Methods*.

[31] Preacher K. J., Hayes A. F. (2008). Asymptotic and resampling strategies for assessing and comparing indirect effects in multiple mediator models. *Behavior Research Methods*.

[32] Zhao X. S., Lynch J. G., Chen Q. M. (2010). Reconsidering Baron and Kenny: myths and truths about mediation analysis. *Journal of Consumer Research*.

[33] Baron R. M., Kenny D. A. (1986). The moderator-mediator variable distinction in social psychological research: conceptual, strategic, and statistical considerations. *Journal of Personality and Social Psychology*.

[34] MacKinnon D. P., Krull J. L., Lockwood C. M. (2000). Equivalence of the mediation, confounding and suppression effect. *Prevention Science*.

[35] Qin Z., Zhang L., Sun F. (2009). Health related quality of life in early Parkinson’s disease: impact of motor and non-motor symptoms, results from Chinese levodopa exposed cohort. *Parkinsonism and Related Disorders*.

[36] Fereshtehnejad S. M., Shafieesabet M., Farhadi F. (2015). Heterogeneous determinants of quality of life in different phenotypes of Parkinson’s disease. *PLoS One*.

[37] Hinnell C., Hurt C. S., Landau S., Brown R. G., Samuel M. (2012). Nonmotor versus motor symptoms: how much do they matter to health status in Parkinson’s disease?. *Movement Disorders*.

[38] Gallagher D. A., Lees A. J., Schrag A. (2010). What are the most important nonmotor symptoms in patients with Parkinson’s disease and are we missing them?. *Movement Disorders*.

[39] Martinez-Martin P., Rodriguez-Blazquez C., Kurtis M. M., Chaudhuri K. R. (2011). The impact of non-motor symptoms on health-related quality of life of patients with Parkinson’s disease. *Movement Disorders*.

[40] Soleimani M. A., Bastani F., Negarandeh R., Greysen R. (2016). Perceptions of people living with Parkinson’s disease: a qualitative study in Iran. *British Journal of Community Nursing*.

[41] Valkovic P., Harsany J., Hanakova M., Martinkova J., Benetin J. (2014). Nonmotor symptoms in early- and advanced-stage Parkinson’s disease patients on dopaminergic therapy: how do they correlate with quality of life?. *ISRN Neurology*.

[42] Birgersson A. M., Edberg A. K. (2004). Being in the light or in the shade: persons with Parkinson’s disease and their partners’ experience of support. *International Journal of Nursing Studies*.

